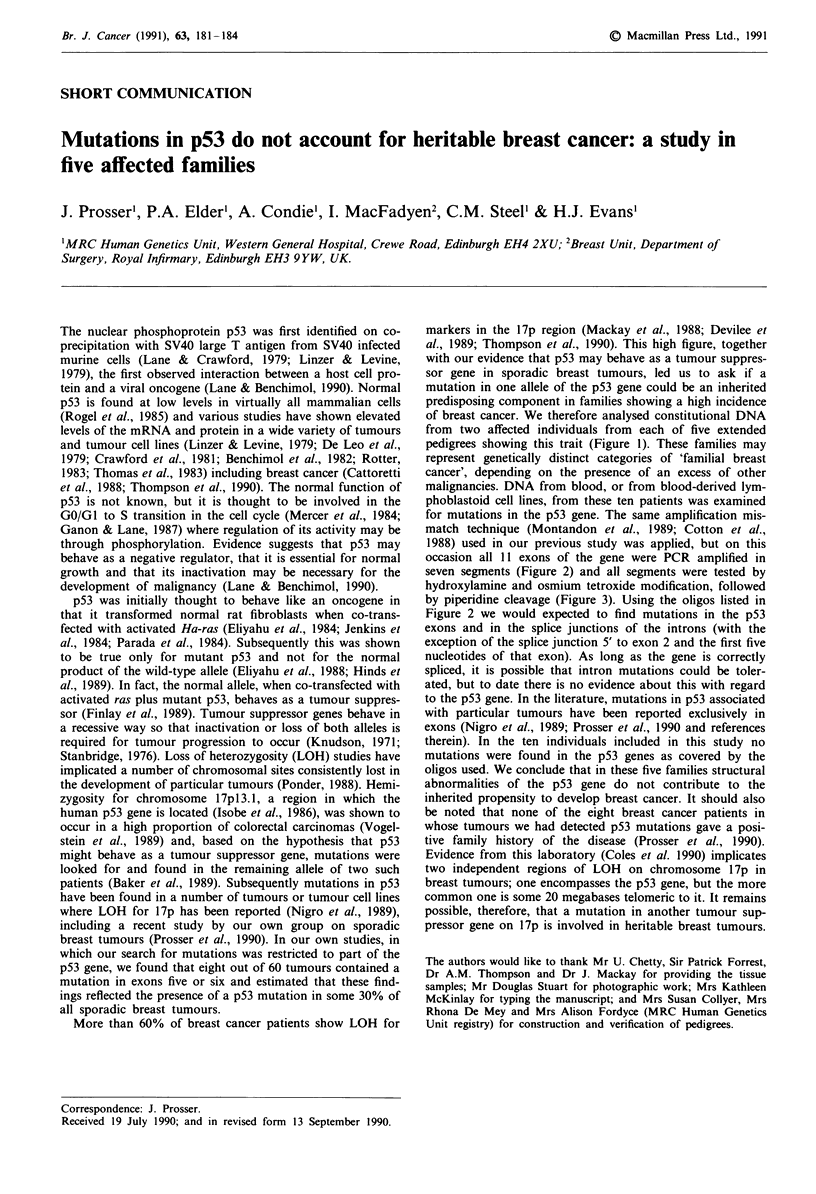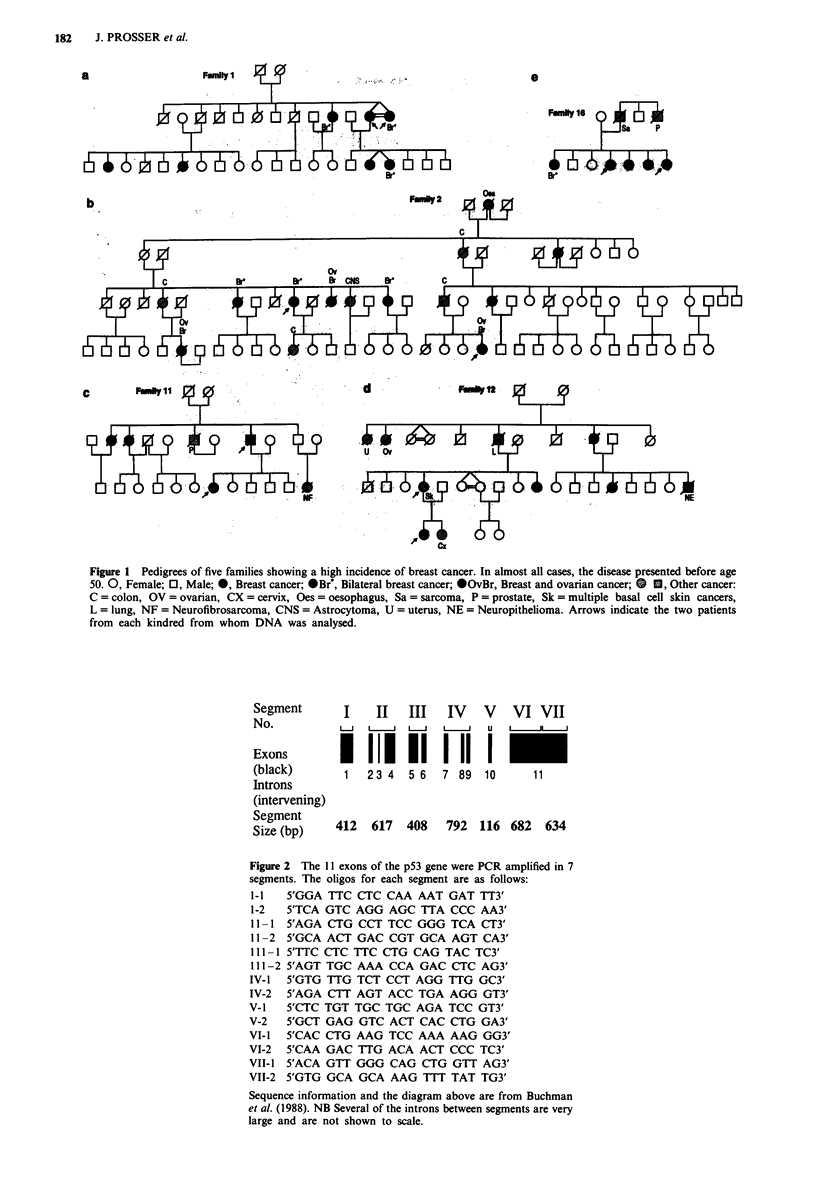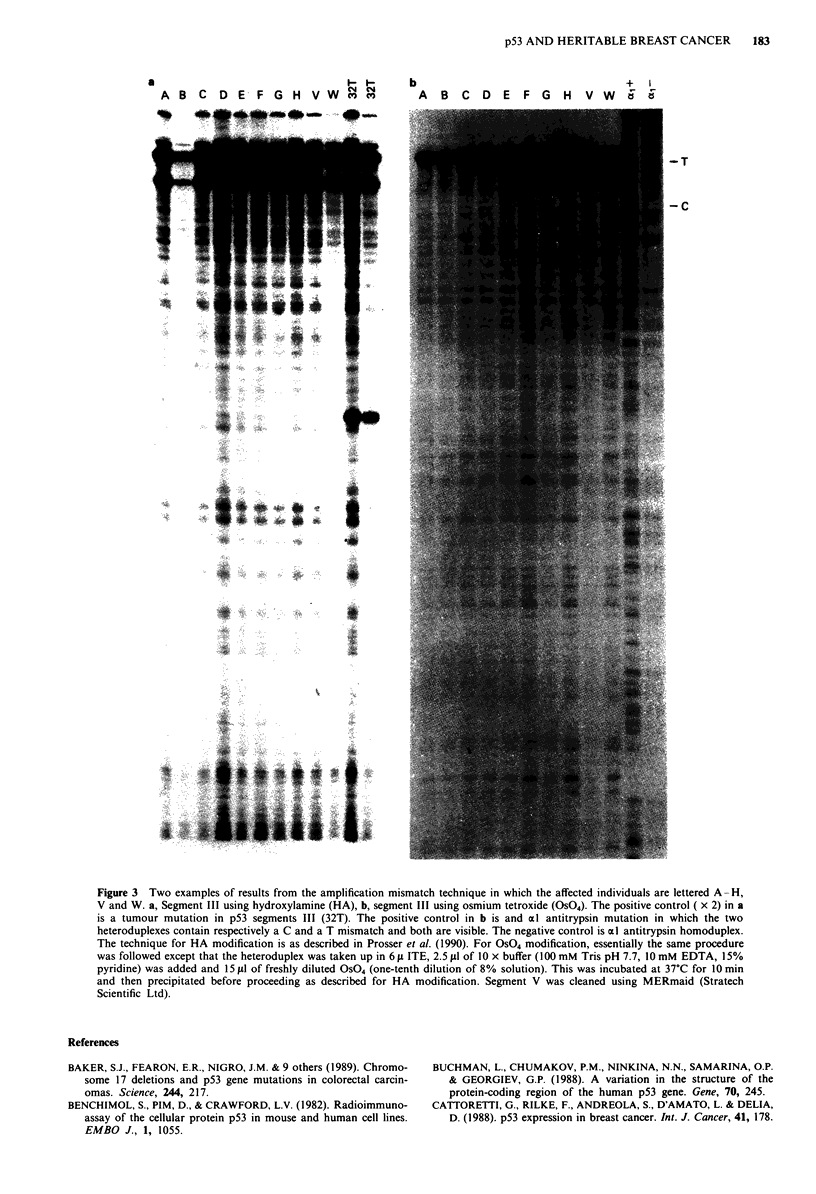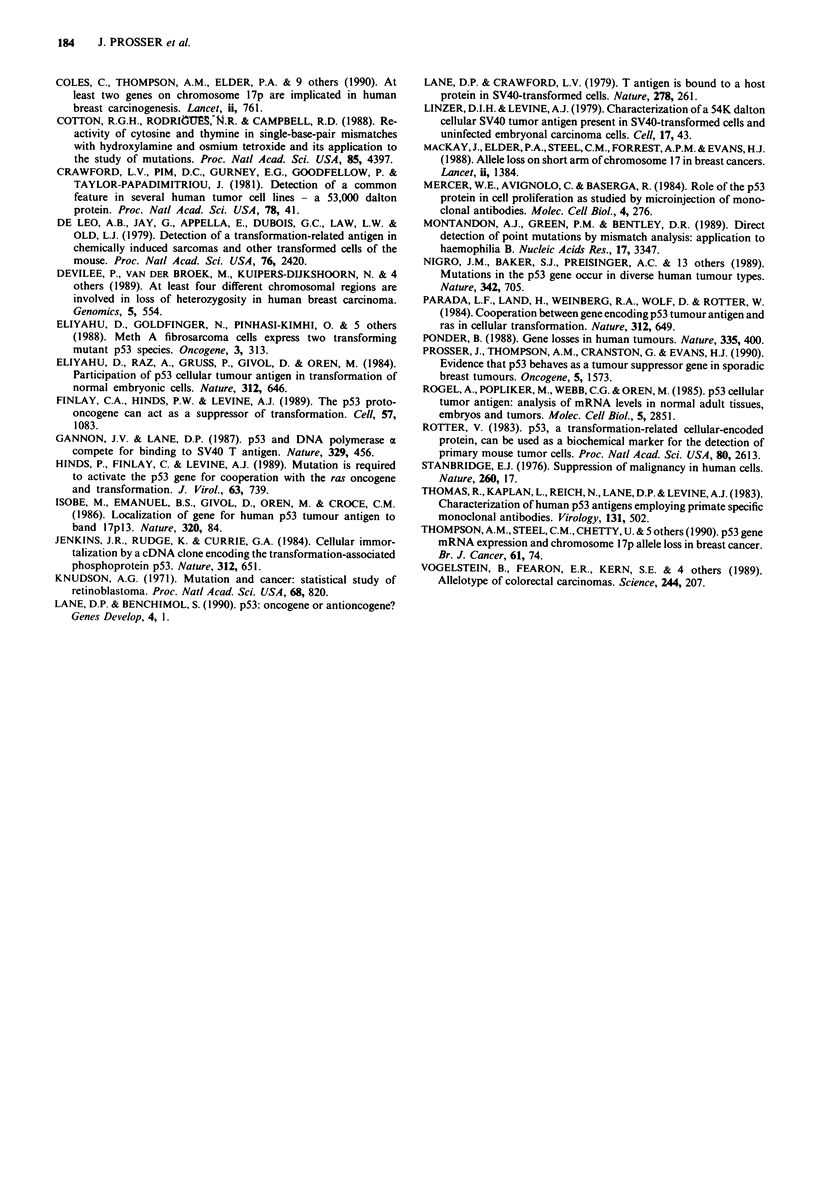# Mutations in p53 do not account for heritable breast cancer: a study in five affected families.

**DOI:** 10.1038/bjc.1991.44

**Published:** 1991-02

**Authors:** J. Prosser, P. A. Elder, A. Condie, I. MacFadyen, C. M. Steel, H. J. Evans

**Affiliations:** MRC Human Genetics Unit, Western General Hospital, Edinburgh, UK.

## Abstract

**Images:**


Br. J. Cancer (1991), 63, 181-184 © Macmillan Press Ltd., 1991

SHORT COMMUNICATION

### Mutations in p53 do not account for heritable breast cancer: a study in five affected families

J. Prosser[1], P.A. Elder[1], A. Condie[1], I. MacFadyen[2], C.M. Steel[1] & H.J. Evans[1]

[1]*MRC Human Genetics Unit, Western General Hospital, Crewe Road, Edinburgh EH4 2XU;* [2]*Breast Unit, Department of Surgery, Royal Infirmary, Edinburgh EH3 9YW, UK.*

The nuclear phosphoprotein p53 was first identified on co-precipitation with SV40 large T antigen from SV40 infected murine cells (Lane & Crawford, 1979; Linzer & Levine, 1979), the first observed interaction between a host cell protein and a viral oncogene (Lane & Benchimol, 1990). Normal p53 is found at low levels in virtually all mammalian cells (Rogel *et al.*, 1985) and various studies have shown elevated levels of the mRNA and protein in a wide variety of tumours and tumour cell lines (Linzer & Levine, 1979; De Leo *et al.*, 1979; Crawford *et al.*, 1981; Benchimol *et al.*, 1982; Rotter, 1983; Thomas *et al.*, 1983) including breast cancer (Cattoretti *et al.*, 1988; Thompson *et al.*, 1990). The normal function of p53 is not known, but it is thought to be involved in the G0/G1 to S transition in the cell cycle (Mercer *et al.*, 1984; Ganon & Lane, 1987) where regulation of its activity may be through phosphorylation. Evidence suggests that p53 may behave as a negative regulator, that it is essential for normal growth and that its inactivation may be necessary for the development of malignancy (Lane & Benchimol, 1990).

p53 was initially thought to behave like an oncogene in that it transformed normal rat fibroblasts when co-transfected with activated *Ha-ras* (Eliyahu *et al.*, 1984; Jenkins *et al.*, 1984; Parada *et al.*, 1984). Subsequently this was shown to be true only for mutant p53 and not for the normal product of the wild-type allele (Eliyahu *et al.*, 1988; Hinds *et al.*, 1989). In fact, the normal allele, when co-transfected with activated *ras* plus mutant p53, behaves as a tumour suppressor (Finlay *et al.*, 1989). Tumour suppressor genes behave in a recessive way so that inactivation or loss of both alleles is required for tumour progression to occur (Knudson, 1971; Stanbridge, 1976). Loss of heterozygosity (LOH) studies have implicated a number of chromosomal sites consistently lost in the development of particular tumours (Ponder, 1988). Hemizygosity for chromosome 17p13.1, a region in which the human p53 gene is located (Isobe *et al.*, 1986), was shown to occur in a high proportion of colorectal carcinomas (Vogelstein *et al.*, 1989) and, based on the hypothesis that p53 might behave as a tumour suppressor gene, mutations were looked for and found in the remaining allele of two such patients (Baker *et al.*, 1989). Subsequently mutations in p53 have been found in a number of tumours or tumour cell lines where LOH for 17p has been reported (Nigro *et al.*, 1989), including a recent study by our own group on sporadic breast tumours (Prosser *et al.*, 1990). In our own studies, in which our search for mutations was restricted to part of the p53 gene, we found that eight out of 60 tumours contained a mutation in exons five or six and estimated that these findings reflected the presence of a p53 mutation in some 30% of all sporadic breast tumours.

More than 60% of breast cancer patients show LOH for markers in the 17p region (Mackay *et al.*, 1988; Devilee *et al.*, 1989; Thompson *et al.*, 1990). This high figure, together with our evidence that p53 may behave as a tumour suppressor gene in sporadic breast tumours, led us to ask if a mutation in one allele of the p53 gene could be an inherited predisposing component in families showing a high incidence of breast cancer. We therefore analysed constitutional DNA from two affected individuals from each of five extended pedigrees showing this trait (Figure 1). These families may represent genetically distinct categories of 'familial breast cancer', depending on the presence of an excess of other malignancies. DNA from blood, or from blood-derived lymphoblastoid cell lines, from these ten patients was examined for mutations in the p53 gene. The same amplification mismatch technique (Montandon *et al.*, 1989; Cotton *et al.*, 1988) used in our previous study was applied, but on this occasion all 11 exons of the gene were PCR amplified in seven segments (Figure 2) and all segments were tested by hydroxylamine and osmium tetroxide modification, followed by piperidine cleavage (Figure 3). Using the oligos listed in Figure 2 we would expected to find mutations in the p53 exons and in the splice junctions of the introns (with the exception of the splice junction 5′ to exon 2 and the first five nucleotides of that exon). As long as the gene is correctly spliced, it is possible that intron mutations could be tolerated, but to date there is no evidence about this with regard to the p53 gene. In the literature, mutations in p53 associated with particular tumours have been reported exclusively in exons (Nigro *et al.*, 1989; Prosser *et al.*, 1990 and references therein). In the ten individuals included in this study no mutations were found in the p53 genes as covered by the oligos used. We conclude that in these five families structural abnormalities of the p53 gene do not contribute to the inherited propensity to develop breast cancer. It should also be noted that none of the eight breast cancer patients in whose tumours we had detected p53 mutations gave a positive family history of the disease (Prosser *et al.*, 1990). Evidence from this laboratory (Coles *et al.* 1990) implicates two independent regions of LOH on chromosome 17p in breast tumours; one encompasses the p53 gene, but the more common one is some 20 megabases telomeric to it. It remains possible, therefore, that a mutation in another tumour suppressor gene on 17p is involved in heritable breast tumours.

The authors would like to thank Mr U. Chetty, Sir Patrick Forrest, Dr A.M. Thompson and Dr J. Mackay for providing the tissue samples; Mr Douglas Stuart for photographic work; Mrs Kathleen McKinlay for typing the manuscript; and Mrs Susan Collyer, Mrs Rhona De Mey and Mrs Alison Fordyce (MRC Human Genetics Unit registry) for construction and verification of pedigrees.

Correspondence: J. Prosser.
Received 19 July 1990; and in revised form 13 September 1990.

**Figure 1** Pedigrees of five families showing a high incidence of breast cancer. In almost all cases, the disease presented before age 50. O, Female; □, Male; ●, Breast cancer; ●Br*, Bilateral breast cancer; ●OvBr, Breast and ovarian cancer; ◍ ▩, Other cancer: C = colon, OV = ovarian, CX = cervix, Oes = oesophagus, Sa = sarcoma, P = prostate, Sk = multiple basal cell skin cancers, L = lung, NF = Neurofibrosarcoma, CNS = Astrocytoma, U = uterus, NE = Neuropithelioma. Arrows indicate the two patients from each kindred from whom DNA was analysed.

**Figure 2** The 11 exons of the p53 gene were PCR amplified in 7 segments. The oligos for each segment are as follows:

| | |
|---|---|
| 1-1 | 5′GGA TTC CTC CAA AAT GAT TT3′ |
| 1-2 | 5′TCA GTC AGG AGC TTA CCC AA3′ |
| 11-1 | 5′AGA CTG CCT TCC GGG TCA CT3′ |
| 11-2 | 5′GCA ACT GAC CGT GCA AGT CA3′ |
| 111-1 | 5′TTC CTC TTC CTG CAG TAC TC3′ |
| 111-2 | 5′AGT TGC AAA CCA GAC CTC AG3′ |
| IV-1 | 5′GTG TTG TCT CCT AGG TTG GC3′ |
| IV-2 | 5′AGA CTT AGT ACC TGA AGG GT3′ |
| V-1 | 5′CTC TGT TGC TGC AGA TCC GT3′ |
| V-2 | 5′GCT GAG GTC ACT CAC CTG GA3′ |
| VI-1 | 5′CAC CTG AAG TCC AAA AAG GG3′ |
| VI-2 | 5′CAA GAC TTG ACA ACT CCC TC3′ |
| VII-1 | 5′ACA GTT GGG CAG CTG GTT AG3′ |
| VII-2 | 5′GTG GCA GCA AAG TTT TAT TG3′ |

Sequence information and the diagram above are from Buchman *et al.* (1988). NB Several of the introns between segments are very large and are not shown to scale.

**Figure 3** Two examples of results from the amplification mismatch technique in which the affected individuals are lettered A–H, V and W. **a**, Segment III using hydroxylamine (HA), **b**, segment III using osmium tetroxide ($OsO_4$). The positive control ( × 2) in **a** is a tumour mutation in p53 segments III (32T). The positive control in **b** is and α1 antitrypsin mutation in which the two heteroduplexes contain respectively a C and a T mismatch and both are visible. The negative control is α1 antitrypsin homoduplex. The technique for HA modification is as described in Prosser *et al.* (1990). For $OsO_4$ modification, essentially the same procedure was followed except that the heteroduplex was taken up in 6 µ ITE, 2.5 µl of 10 × buffer (100 mM Tris pH 7.7, 10 mM EDTA, 15% pyridine) was added and 15 µl of freshly diluted $OsO_4$ (one-tenth dilution of 8% solution). This was incubated at 37°C for 10 min and then precipitated before proceeding as described for HA modification. Segment V was cleaned using MERmaid (Stratech Scientific Ltd).

### References

BAKER, S.J., FEARON, E.R., NIGRO, J.M. & 9 others (1989). Chromosome 17 deletions and p53 gene mutations in colorectal carcinomas. *Science*, **244**, 217.

BENCHIMOL, S., PIM, D., & CRAWFORD, L.V. (1982). Radioimmunoassay of the cellular protein p53 in mouse and human cell lines. *EMBO J.*, **1**, 1055.

BUCHMAN, L., CHUMAKOV, P.M., NINKINA, N.N., SAMARINA, O.P. & GEORGIEV, G.P. (1988). A variation in the structure of the protein-coding region of the human p53 gene. *Gene*, **70**, 245.

CATTORETTI, G., RILKE, F., ANDREOLA, S., D'AMATO, L. & DELIA, D. (1988). p53 expression in breast cancer. *Int. J. Cancer*, **41**, 178.

COLES, C., THOMPSON, A.M., ELDER, P.A. & 9 others (1990). At least two genes on chromosome 17p are implicated in human breast carcinogenesis. *Lancet*, **ii**, 761.

COTTON, R.G.H., RODRIGUES, N.R. & CAMPBELL, R.D. (1988). Reactivity of cytosine and thymine in single-base-pair mismatches with hydroxylamine and osmium tetroxide and its application to the study of mutations. *Proc. Natl Acad. Sci. USA*, **85**, 4397.

CRAWFORD, L.V., PIM, D.C., GURNEY, E.G., GOODFELLOW, P. & TAYLOR-PAPADIMITRIOU, J. (1981). Detection of a common feature in several human tumor cell lines – a 53,000 dalton protein. *Proc. Natl Acad. Sci. USA*, **78**, 41.

DE LEO, A.B., JAY, G., APPELLA, E., DUBOIS, G.C., LAW, L.W. & OLD, L.J. (1979). Detection of a transformation-related antigen in chemically induced sarcomas and other transformed cells of the mouse. *Proc. Natl Acad. Sci. USA*, **76**, 2420.

DEVILEE, P., VAN DER BROEK, M., KUIPERS-DIJKSHOORN, N. & 4 others (1989). At least four different chromosomal regions are involved in loss of heterozygosity in human breast carcinoma. *Genomics*, **5**, 554.

ELIYAHU, D., GOLDFINGER, N., PINHASI-KIMHI, O. & 5 others (1988). Meth A fibrosarcoma cells express two transforming mutant p53 species. *Oncogene*, **3**, 313.

ELIYAHU, D., RAZ, A., GRUSS, P., GIVOL, D. & OREN, M. (1984). Participation of p53 cellular tumour antigen in transformation of normal embryonic cells. *Nature*, **312**, 646.

FINLAY, C.A., HINDS, P.W. & LEVINE, A.J. (1989). The p53 proto-oncogene can act as a suppressor of transformation. *Cell*, **57**, 1083.

GANNON, J.V. & LANE, D.P. (1987). p53 and DNA polymerase α compete for binding to SV40 T antigen. *Nature*, **329**, 456.

HINDS, P., FINLAY, C. & LEVINE, A.J. (1989). Mutation is required to activate the p53 gene for cooperation with the *ras* oncogene and transformation. *J. Virol.*, **63**, 739.

ISOBE, M., EMANUEL, B.S., GIVOL, D., OREN, M. & CROCE, C.M. (1986). Localization of gene for human p53 tumour antigen to band 17p13. *Nature*, **320**, 84.

JENKINS, J.R., RUDGE, K. & CURRIE, G.A. (1984). Cellular immortalization by a cDNA clone encoding the transformation-associated phosphoprotein p53. *Nature*, **312**, 651.

KNUDSON, A.G. (1971). Mutation and cancer: statistical study of retinoblastoma. *Proc. Natl Acad. Sci. USA*, **68**, 820.

LANE, D.P. & BENCHIMOL, S. (1990). p53: oncogene or antioncogene? *Genes Develop*, **4**, 1.

LANE, D.P. & CRAWFORD, L.V. (1979). T antigen is bound to a host protein in SV40-transformed cells. *Nature*, **278**, 261.

LINZER, D.I.H. & LEVINE, A.J. (1979). Characterization of a 54K dalton cellular SV40 tumor antigen present in SV40-transformed cells and uninfected embryonal carcinoma cells. *Cell*, **17**, 43.

MACKAY, J., ELDER, P.A., STEEL, C.M., FORREST, A.P.M. & EVANS, H.J. (1988). Allele loss on short arm of chromosome 17 in breast cancers. *Lancet*, **ii**, 1384.

MERCER, W.E., AVIGNOLO, C. & BASERGA, R. (1984). Role of the p53 protein in cell proliferation as studied by microinjection of monoclonal antibodies. *Molec. Cell Biol.*, **4**, 276.

MONTANDON, A.J., GREEN, P.M. & BENTLEY, D.R. (1989). Direct detection of point mutations by mismatch analysis: application to haemophilia B. *Nucleic Acids Res.*, **17**, 3347.

NIGRO, J.M., BAKER, S.J., PREISINGER, A.C. & 13 others (1989). Mutations in the p53 gene occur in diverse human tumour types. *Nature*, **342**, 705.

PARADA, L.F., LAND, H., WEINBERG, R.A., WOLF, D. & ROTTER, W. (1984). Cooperation between gene encoding p53 tumour antigen and ras in cellular transformation. *Nature*, **312**, 649.

PONDER, B. (1988). Gene losses in human tumours. *Nature*, **335**, 400.

PROSSER, J., THOMPSON, A.M., CRANSTON, G. & EVANS, H.J. (1990). Evidence that p53 behaves as a tumour suppressor gene in sporadic breast tumours. *Oncogene*, **5**, 1573.

ROGEL, A., POPLIKER, M., WEBB, C.G. & OREN, M. (1985). p53 cellular tumor antigen: analysis of mRNA levels in normal adult tissues, embryos and tumors. *Molec. Cell Biol.*, **5**, 2851.

ROTTER, V. (1983). p53, a transformation-related cellular-encoded protein, can be used as a biochemical marker for the detection of primary mouse tumor cells. *Proc. Natl Acad. Sci. USA*, **80**, 2613.

STANBRIDGE, E.J. (1976). Suppression of malignancy in human cells. *Nature*, **260**, 17.

THOMAS, R., KAPLAN, L., REICH, N., LANE, D.P. & LEVINE, A.J. (1983). Characterization of human p53 antigens employing primate specific monoclonal antibodies. *Virology*, **131**, 502.

THOMPSON, A.M., STEEL, C.M., CHETTY, U. & 5 others (1990). p53 gene mRNA expression and chromosome 17p allele loss in breast cancer. *Br. J. Cancer*, **61**, 74.

VOGELSTEIN, B., FEARON, E.R., KERN, S.E. & 4 others (1989). Allelotype of colorectal carcinomas. *Science*, **244**, 207.